# Urinary Schistosomiasis among Children in Murbai and Surbai Communities of Ardo-Kola Local Government Area, Taraba State, Nigeria

**DOI:** 10.1155/2016/9831265

**Published:** 2016-12-14

**Authors:** R. S. Houmsou, H. Agere, B. E. Wama, J. B. Bingbeng, E. U. Amuta, S. L. Kela

**Affiliations:** ^1^Department of Biological Sciences, Taraba State University, Jalingo, Nigeria; ^2^Department of Biological Sciences, Federal University Wukari, Taraba State, Nigeria; ^3^Department of Biology, College of Education, Zing, Taraba State, Nigeria; ^4^Department of Biological Sciences, University of Agriculture Makurdi, Benue State, Nigeria; ^5^Department of Biological Sciences, Federal University Kashere, Gombe State, Nigeria

## Abstract

*Background*. This cross-sectional study was conducted to determine the prevalence, intensity of infection, and risk factors associated with urinary schistosomiasis among children in Murbai and Surbai communities of Ardo-Kola Local Government Area (LGA), Taraba State, Nigeria.* Methods*. Urine samples were analysed by the standard filtration technique using 10 ml syringe, Swinnex polypropylene filter holder (13 mm diameter), and polycarbonate membrane filters (12 *μ*m porosity). Sociodemographic data and water contact activities were collated from children using structured questionnaires.* Results*. A point prevalence of 58.54% was reported out of the urine samples examined. Males were significantly more infected than their female counterparts (71.15% versus 43.66%, *χ* ^2^ = 89.12, *p* = 0.000). The age-related prevalence showed 6–10 and 11–15 years significantly infected with 78.70% and 73.02%, respectively (*χ* ^2^ = 89.12, *p* = 0.000). Light intensity of infection, 62.51%, was significantly higher than heavy intensity, 37.48%, among the infected children (*χ* ^2^ = 365.8, *p* = 0.000). Water contact activities such as fishing (OR = 4.01, CI = 3.04–5.61, *p* = 0.000), rice farming (OR = 4.01, CI = 2.96–5.36, *p* = 0.000), and dry season farming (OR = 4.78, CI = 3.68–6.22, *p* = 0.000) were the risk factors exposing children to infection in the area.* Conclusion*. There is an urgent need to undertake a large scale deworming control programme using praziquantel in the area.

## 1. Introduction

Human schistosomiasis is a waterborne parasitic infection caused by species of* Schistosoma*, namely,* Schistosoma haematobium *(*S. haematobium*),* S. mansoni*,* S. japonicum*,* S. mekongi*, and* S. intercalatum* [1]. Worldwide, about 779 million are estimated to be at risk of infection with 249 million infected [2, 3]. The heavy burden is carried by sub-Saharan Africa where an estimated 224 million suffer the malignant effects of the disease with an estimated 280,000 death toll every year mostly among the rural inhabitants [3].

In sub-Saharan Africa, Nigeria carries the heaviest burden with an estimated 29 million cases of infection [4, 5]. Both urinary and intestinal schistosomiasis exist in Nigeria [6–9], but urinary schistosomiasis is more widespread than intestinal schistosomiasis with varying prevalence across the country [6, 10–15]. The disease is transmitted by the group of planorbid fresh water snails of the genus* Bulinus* found around sources of water such as streams, slow flowing rivers, ponds, and irrigation canals where rural inhabitants rely on for their recreational, occupational, domestic, and agricultural activities.

During the 65th World Health Assembly, it was advocated that Member States should intensify control intervention and initiation of elimination programmes for schistosomiasis. These still remain a dream for several countries in sub-Saharan Africa particularly Nigeria where the coverage for the preventive chemotherapy for schistosomiasis is 4.0% [16]. The nonimplementation of the policies is impeded by the lack of political commitment, lack of public health infrastructures, and the necessary resources to initiate and sustain control programmes across the country.

Taraba State is well-known for its diverse relief made of mountainous and plain areas. Many of the plain areas are places of intense agricultural activities. These plain areas are surrounded by streams, rivers, and ponds where inhabitants depend on ignorantly neglecting the health implications. The indigenous people in the state depend on fishing and agricultural activities such as rice and irrigation farming with complete dependence on the existing natural water sources. There is a paucity of information on the distribution of urinary schistosomiasis in the state. Moreover, an extensive mapping programme is required to facilitate the scaling-up of the much needed preventive chemotherapy intervention in rural communities. Thus, this study was conducted to investigate urinary schistosomiasis in relation to epidemiological factors, as well as the associated risk factors that predispose children to infection in two riverine communities (Murbai and Surbai) of Ardo-Kola Local Government Area, Taraba State, Nigeria.

## 2. Materials and Methods

### 2.1. Study Area

**Figure 1:**
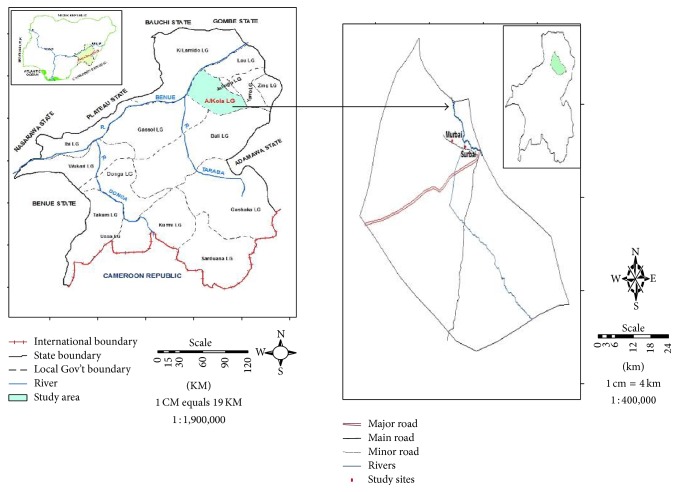
Map of study area.

The geographical positions of the areas are Murbai (08°54′N; 11°88′E) and Surbai (08°54′N; 11°15′E). The Local Government Area is bounded to the North by Jalingo, Lau, and Yorro Local Government Areas; to the West by Karim-Lamido LGA; and to the South and South West by Bali and Gassol LGAs, respectively (Figure 1). The area is inhabited mainly by indigenous people “the jukun kona” that are peasant farmers that practise both rainy and dry season farming. The climate of the area is tropical with typical savannah vegetation. The area is traversed by streams, rivers, and ponds which the inhabitants rely on for their daily chores activities. The rainy season in the area starts from May to October, while the dry season starts from November to April. The choice of the area was based on several complaints laid to the primary health care officials by children's parents on painful urination and bloody urine. The presence of streams and ponds in the area coupled with the complete lack of epidemiological data prompted the conduct of this study.

### 2.2. Subjects and Inclusion and Exclusion Criteria

The study enrolled pre- and school-aged children that were residents of the two communities. All children who consented and whose parents agreed were included in the study, while those that were physically sick and females that were on their menstrual periods were excluded.

### 2.3. Study Design, Sample Size Calculation, and Sampling Procedures

The design of the study is cross-sectional in nature cutting across sociodemographic and predisposing factors of the children in the area.

The sample size was calculated using the following formula at 95% confidence interval [17]:

(1) $$\begin{array}{c} N=\frac{Z_{\alpha /2*P*\left(1-P\right)*D}^{2}}{E^{2}}, \end{array}$$

where *P* is assumed prevalence of urinary schistosomiasis, which was about 40% when we used the primary health care records. *E* is precision (margin error), which is 10% of the assumed prevalence. *Z*_*α*/2_ = 1.96, which is the normal deviate for two-tailed alternative hypothesis. *D* = 1, which is the design effect reflecting the simple random sampling procedure used for children enrolment.

The calculated children population size *N* was 576. Due to the difference in population size of the communities, 10% of the sample size calculated about (58) was required to be added during sampling in Murbai community. After data collection, these were sorted and we arrived at 541 in Surbai and 612 in Murbai communities, respectively.

### 2.4. Focus Group Discussions (FGDs) and Questionnaire Administration

Prior to the administration of questionnaires, focus group discussions were held with parents/guardians, children's mothers, communities' heads, and primary health care and schools officials. The interviews focused on whether there was a launch of a mass drug administration campaign of praziquantel in the previous years. The research team also interviewed the inhabitants on their water contact activities in the area.

During administration of questionnaires, children were grouped into three categories: 1–5 years, 6–10 years, and 11–15 years old. Mothers/guardians of children aged 1–5 years and children aged 6–10 years were interviewed individually using the local dialect during which the interpreter concomitantly filled the questionnaire of each child. The older children (11–15 years) were given questionnaires to be filled individually under the guide of a trained teacher on the subject matter. Whenever there was misunderstanding of some questions, these were translated into the local dialect for better understanding. Administered questionnaires collated information on sociodemographic factors such as age, sex, educational level, and occupation of parents and water contact activities such as swimming, fishing, playing/bathing, irrigation, and rice farming.

### 2.5. Urine Sample Collection and Laboratory Investigations

About 20 ml of urine was collected from each of the enrolled child using labelled universal bottles. Samples were collected between 10:00–14:00 hours of the day, preserved in the cooler using ice pack, and transported to the laboratory within 30 minutes.

In the laboratory, urine specimens were processed by the standard filtration technique using 10 ml syringe, Swinnex polypropylene filter holder (13 mm diameter), and polycarbonate membrane filters (12 *μ*m porosity) (Sterlitech Corporation, Kent, USA). Positive samples were reported and classified as light intensity of infection (1–49 eggs/10 ml of urine) and heavy intensity of infection (>50 eggs/10 ml of urine) [18].

### 2.6. Data Entry and Analysis

Collated data were entered and sorted into Microsoft Excel 2010 and exported into SPSS IBM version 20 for data analysis. Chi-square (*χ* ^2^) test was used to compare infection level as well as intensity of infection between communities, age, sex, educational level, and occupation of children parents. Logistic regression was used to assess possible relationship between schistosomiasis level and children predisposing factors. *p* ≤ 0.05 was used as significance level.

## 3. Results

The occurrence of urinary schistosomiasis in relation to age, sex, and communities surveyed is shown in Table 1. The overall infection level was 58.54% (675/1153) among the children examined. The infection varied significantly between male, 71.15% (444/624), and female, 43.66% (231/529) (*χ* ^2^ = 89.12, *p* = 0.000), as well as between age groups with the 6–10 years, 78.70% (436/554), and 11–15 years, 73.02% (176/241), having the highest level of infection (*χ* ^2^ = 360.88; *p* = 0.000). Communities related infection reported that children in Surbai, 66.72% (361/541), were infected compared to those in Murbai, 51.30% (314/612) (*χ* ^2^ = 28.13, *p* = 0.000).

**Figure 2:**
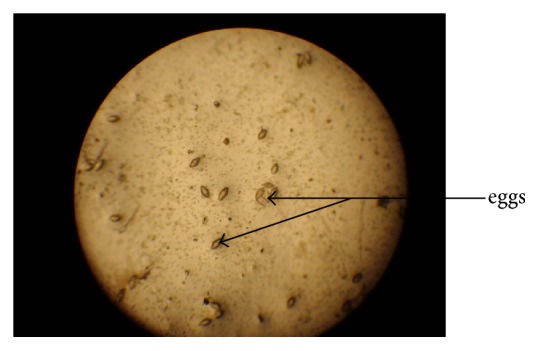
Light intensity of infection (1–49 eggs/10 ml of urine) (×10 objective) (field study, 2015).

**Figure 3:**
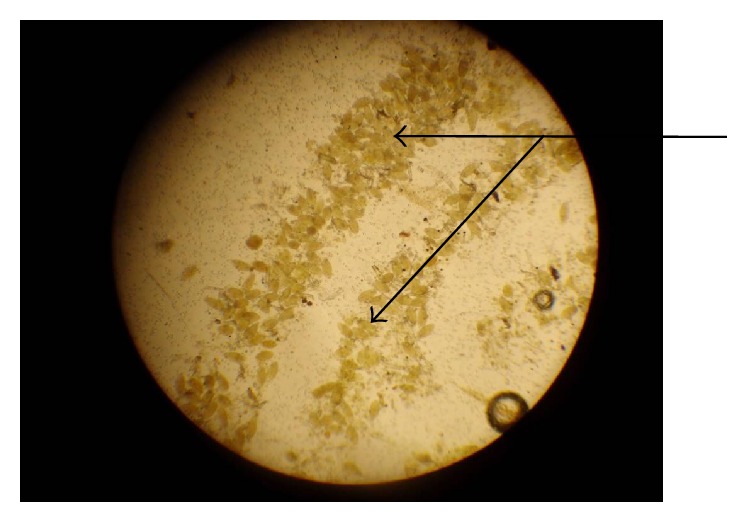
Heavy intensity of infection (>50 eggs/10 ml of urine) (×10 objective) (field study, 2015).

Table 2 shows the trend of intensity of* S. haematobium* eggs among the infected children. Light intensity of infection (1–49 eggs/10 ml of urine) (Figure 2), 62.51% (422/675), was significantly reported compared to heavy intensity of infection (>50 eggs/10 ml of urine) (Figure 3), 37.48% (253/675) (*χ* ^2^ = 96.99, *p* = 0.000). The sex trend showed that female significantly carried the burden of light intensity of infection (70.12%), while male carried the highest burden of heavy intensity of infection (41.44%) (*χ* ^2^ = 96.99; *p* = 0.000). The age-related intensity of infection showed that the age group 1–5 years significantly carried the burden of light intensity of infection (85.71%), while the older age group 11–15 years carried the highest burden of heavy intensity of infection (44.31%) (*χ* ^2^ = 369.69; *p* = 0.000).

The risk factors exposing the children to urinary schistosomiasis in Murbai and Surbai communities of Taraba State, Nigeria, are shown in Table 3. Children involved in dry season farming (irrigation) [71.01%, OR = 4.789, CI (3.685–6.224), *p* = 0.000]; fishing [66.36%, OR = 4.013; CI (3.004–5.361), *p* = 0.000]; and rice farming [66.21%, OR = 4.010; CI (2.996–5.368), *p* = 0.000] were more exposed to urinary schistosomiasis.

## 4. Discussion

Schistosomiasis has received little attention compared to other neglected tropical diseases in Nigeria. This is because the disease does not cause immediate chronic effects on the infected individuals. All these years, efforts made to control the disease have not significantly reduced the burden from the affected people that are still despaired and suffering from its adverse effects. The overall infection level (58.54%) reported in this present study showed hyperendemicity of urinary schistosomiasis in the area. This corroborates the World Health Organization classification of schistosomiasis endemicity [18]. This hyperendemicity is worrisome because, during our focus group discussion with the community leaders and primary health care and schools officials, no praziquantel distribution campaign has ever been conducted in the area. This noninitiation and nonexistence of a control activity in the area will definitely hamper the efforts and goals set by the resolution WHA 54.19 of the World Health Assembly [19].

The hyperendemicity of the disease in the two communities is attributed to the intensified water contact activities of the inhabitants such as washing of utensils, bathing, and swimming in cercariae infested streams and ponds. The agricultural activities such as the dry season farm practice using ponds water and cultivation of rice in swampy areas are common among the inhabitants. These practices are as well contributory factors to the hyperendemicity of the disease. More so, the complete deprivation of these communities of potable water despite their proximity to the Taraba State capital, Jalingo, has also coerced them to depend on these infested water bodies. There is need for immediate concerted efforts among the stakeholders to initiate a control programme in the area to avoid an outbreak. It has been found that children in those communities visit neighbouring villages for their recreational activities, while the older ones go to secondary schools located around the periphery of the capital city where they have access to other water bodies.

Urinary schistosomiasis occurrence in this study is corroborated by findings reported in other parts of rural Nigeria: 55.0% in some rural areas of Guma LGA, Benue State [11]; 53.8% in some rural areas of Abia State [12]; 58.1% in a rural community near Abeokuta, Ogun State [13]; and 61.5% in communities living around the Erinle and Eko-Ende dams, Osun State [20]. The hyperendemicity in all these communities reflects the common behavioural water contact activities of the inhabitants through either their daily chores or recreational activities and agricultural engagements. This is pertinent because even if preventive chemotherapy is given to all village dwellers, the dearth of potable water will still constrain people to go into contact with cercariae infested water bodies. The present finding contrasts report of the Federal Ministry of Health which found low endemicity of schistosomiasis (5.6%) in nine LGAs of Taraba State [21]. Schistosomiasis infection level in the present study was significantly higher than findings in other parts of the state, 10.1% and 15.5% in Gashaka and Bali LGAs, respectively [6, 22], as well as 15.3% in a rural community of southwestern Ebonyi State [23] and 8.3% among Hausa communities of five LGAs in Kano State [24]. The infection level found in this study is also higher than 49.0% and 44.3% reported in Biase, Cross-River State [25], and Toto LGA, Nasarawa State [26], respectively, which are both neighbouring states to Taraba State.

Compared to other rural areas of African countries, the infection level reported in this study is similar to those reported in Senegal and Sudan [26, 27] but higher than reports from Ethiopia [28].

Most children had light intensity of infection, but this was higher among females and children aged 1–10 years. Conversely, a fewer portion of the children carried the burden of heavy intensity of infection in the area with male and children aged 11–15 years mostly affected. This seems to be the trend in most urinary schistosomiasis affected areas. This phenomenon has not been clearly understood and explained in most studies and leaves insinuation among researchers. The occurrence of light and heavy intensities of infection might depend on the children immune system build-up or the frequency of their water contact activities with cercariae infested water bodies. Such trend has been reported in Nigeria and other endemic countries with no clear explanation [10, 11, 21, 29, 30]. The proportion of the heavy intensity observed among the male and older children portrays serious morbidity. If not treated, this might lead to further complications such as obstructive uropathy, calcified bladder, granuloma reactions to eggs in the mucosa of the urogenital system, and cancer of the bladder at older age [1, 31].

Activities such as fishing, rice farming, and dry season farming seem to predispose children to infection in the area. These activities are the mainstay and were found to be the norm in most Nigerian rural population with absence of safe recreational and potable water. Previous studies reported the contributory role of poor socioeconomical and occupational (fishing and farming) backgrounds as well as recreational activities (swimming, playing, and laundry) in cercariae infested water sources to be major predisposing factors to schistosomiasis in rural communities [3, 32].

In conclusion, this current study reported a new schistosomiasis hyperendemic focus. This will be added to the baseline data that will help in planning future control strategies. Male children were more infected than their female counterparts. Majority of the children were burdened with light intensity of infection. Activities such as fishing, rice farming, and dry season farming were the predisposing risk factors of children to infection. Due to the high level of infection reported in the area, it is recommended that (i) the government, nongovernmental organizations, and international agencies should embark on immediate mass distribution of praziquantel to the entire members of the communities surveyed; (ii) an annual praziquantel distribution campaign be effectively and efficiently undertaken to control the disease in these communities; (iii) strategies such as provision of potable water and sanitation be implemented; (iv) health education and promotion using the local dialect for behavioural changes in the areas are highly advocated; and (v) snails control through the use of environmental friendly molluscicide is advocated. There should be a more political commitment through the creation of a state steering committee. This should involve all stakeholders (government, universities, and other organizations) to promote a state plan of action for integrated control of neglected tropical diseases.

Though urine filtration using polycarbonate membrane filters is the standard field technique for* S. haematobium *eggs recovery, this study had a major limitation which is the single screening of urine specimen from the children. The collection of at least two urine samples on two consecutive days from the children examined would have optimized the recovery of eggs from filters and better estimated the infection level therefore reflecting the true prevalence in the areas.

## Figures and Tables

**Table 1:** Urinary schistosomiasis in relation to communities, age, and sex of children in Surbai and Murbai communities, Taraba State, Nigeria.

	Urinary schistosomiasis (%)		*χ* ^2^	*p value*
	Examined	Positive		
*Variables*				
Overall	1153	675 (58.54)		
Communities			28.13	*0.000*
Murbai	612	314 (51.30)		
Surbai	541	361 (66.72)		
Sex			89.12	*0.000*
Male	624	444 (71.15)		
Female	529	231 (43.66)		
Age (years)			360.88	*0.000*
[1–5]	358	63 (17.59)		
[6–10]	554	436 (78.70)		
[11–15]	241	176 (73.02)		

**Table 2:** Intensity of *Schistosoma haematobium* eggs among infected children in Murbai and Surbai communities, Taraba State, Nigeria.

	Intensity of infection (eggs/10 ml of urine) (%)		Total	*χ* ^2^	*p*
	1–49 eggs	>50 eggs			
Overall	422 (62.51)	253 (37.48)	675		
Sex				96.99	*0.000*
Male	260 (58.55)	184 (41.44)	444		
Female	162 (70.12)	69 (29.87)	231		
Age (years)				369.69	*0.000*
[1–5]	54 (85.71)	9 (14.28)	63		
[6–10]	270 (61.92)	166 (38.07)	436		
[11–15]	98 (55.68)	78 (44.31)	176		

**Table 3:** Risk factors exposing children to urinary schistosomiasis in Murbai and Surbai communities of Taraba State, Nigeria.

	Urinary schistosomiasis (%)		OR	[CI, 95%]	*p* value
	Negative	Positive			
*Variables*					
Swimming			0.75	[0.129–3.864]	0.685
(i) Yes	476 (41.49)	671 (58.50)			
(ii) No	2 (33.33)	4 (66.77)			
Fishing			4.013	[3.004–5.361]	0.000
(i) Yes	297 (33.63)	586 (66.36)			
(ii) No	181 (67.00)	89 (33.00)			
Swimming & fishing			3.286	[2.508–4.306]	0.000
(i) Yes	283 (33.65)	558 (66.35)			
(ii) No	195 (62.50)	117 (37.50)			
Rice farming			4.010	[2.996–5.368]	0.000
(i) Yes	300 (33.78)	588 (66.21)			
(ii) No	178 (67.16)	87 (32.83)			
Dry season farming (irrigation)			4.789	[3.685–6.224]	0.000
(i) Yes	222 (28.98)	544 (71.01)			
(ii) No	256 (66.14)	131 (33.85)			
